# Eosinophilic Granulomatosis with Polyangiitis Presenting with Intermittent Claudication

**DOI:** 10.31662/jmaj.2025-0199

**Published:** 2025-12-05

**Authors:** Koichi Bamba, Daisuke Fujishiro, Tomoki Kawahata, Yuki Kamikokura, Tsubasa Ono, Yuta Ikechi, Kitaru Tanaka, Kensaku Okamoto, Yuichi Makino, Hiroshi Nomoto

**Affiliations:** 1 Division of Endocrinology, Metabolism, and Rheumatology, Department of Internal Medicine, Asahikawa Medical University, Asahikawa, Japan; 2 Department of Diagnostic Pathology, Asahikawa Medical University Hospital, Asahikawa, Japan; 3 Center for Medical Education and Regional Symbiosis, Asahikawa Medical University, Asahikawa, Japan

**Keywords:** eosinophilic granulomatosis with polyangiitis, anti-neutrophil cytoplasmic antibody-associated vasculitis, intermittent claudication, muscle pain, creatine kinase, magnetic resonance imaging

## Abstract

Intermittent claudication (IC), characterized by lower limb weakness and pain during walking that resolves with rest, is most commonly caused by lumbar spinal canal stenosis or peripheral arterial disease. We report a unique case of eosinophilic granulomatosis with polyangiitis (EGPA) presenting with IC. An 80-year-old woman presented with fever, muscle pain, muscle weakness, and IC. She had a history of bronchial asthma and allergic rhinitis. Laboratory tests revealed eosinophilia (4,212 /μL) and elevated creatine kinase levels (505 U/L). Magnetic resonance imaging (MRI) with short tau inversion recovery sequences demonstrated diffuse high-signal intensity across multiple skeletal muscles. A biopsy specimen of the right biceps brachii, taken from a high-signal area identified on MRI, showed inflammatory cell infiltration, primarily composed of eosinophils and lymphocytes in the blood vessel walls. The patient was diagnosed with EGPA-associated skeletal muscle involvement and treated with a moderate dose of glucocorticoids, resulting in significant clinical improvement.

This case highlights that EGPA can present with IC, a rare manifestation of the disease, and underscores the diagnostic value of MRI in such cases. MRI may be a useful tool for localizing biopsy sites in patients with EGPA-related muscle damage. Clinicians should consider anti-neutrophil cytoplasmic antibody-associated vasculitis, including EGPA, in patients presenting with IC and systemic symptoms, and initiate early diagnostic and therapeutic interventions.

## Introduction

Eosinophilic granulomatosis with polyangiitis (EGPA) is a type of anti-neutrophil cytoplasmic antibody (ANCA)-associated vasculitis characterized by systemic necrotizing vasculitis and eosinophilic infiltration in patients with asthma or allergic rhinitis ^(1), (2)^. EGPA frequently causes muscle pain and weakness; however, intermittent claudication (IC) is rare. Here, we describe a case of EGPA presenting with IC and skeletal muscle damage, in which magnetic resonance imaging (MRI) helped identify the biopsy site for diagnosis.

## Case Report

An 80-year-old woman with a history of bronchial asthma and allergic rhinitis experienced muscle pain in both thighs three months before admission, which later spread to her shoulders and upper arms. She developed a fever of 38°C and IC. A few days later, she presented to our emergency department with muscle weakness, primarily affecting both lower legs, and was admitted.

On physical examination, she had spontaneous pain and tenderness in the biceps, triceps, and quadriceps bilaterally. Manual muscle testing yielded a score of four in both the upper and lower limbs. She had no abdominal pain or rash, and the bilateral dorsalis pedis arteries were palpable. Her IC was unaffected by postural changes, and she could walk only approximately 6 m because of leg pain.

Laboratory tests revealed eosinophilia (4,212 /μL), elevated creatine kinase levels (CK, 505 U/L), C-reactive protein (2.25 mg/dL), and immunoglobulin E (5,024 IU/mL). CK-myoglobin, electrolytes, and thyroid function were normal. As summarized in Table 1, immunological tests showed weakly positive antinuclear antibodies (speckled pattern, 1:40), while all disease-specific autoantibodies were negative. Rheumatoid factor was positive (160.9 IU/mL); however, the absence of joint symptoms and the lack of inflammatory findings on joint ultrasonography made rheumatoid arthritis unlikely.

**Table 1:** Laboratory Data on Admission.

Variable	Reference range	Patient’s value	Measurement unit
Ig G	861.0 - 1747.0	1268.0	mg/dL
Ig A	93.00 - 393.0	291.6	mg/dL
Ig M	50.00 - 269.0	58.4	mg/dL
Ig E	≤ 232	5024	IU/mL
CH50	30.00 - 45.0	52.3	U/mL
C3	73.00 - 138.0	104.3	mg/dL
C4	11.00 - 31.0	25.2	mg/dL
RF-IgM	< 15.0	160.9	IU/mL
Anti-CCP Ab	< 4.5	< 0.5	U/mL
Antinuclear Antibody	< 40	40	Times
SPECKLED	< 40	40	Times
Anti-ds-DNA Ab	< 10.0	2.5	IU/mL
Anti-Sm Ab	< 7.0	< 7.0	U/mL
Anti-RNP Ab	< 3.5	< 0.7	U/mL
Anti-SS-A Ab	< 7.0	0.7	U/mL
Anti-SS-B Ab	< 7.0	0.4	U/mL
Anti-topoisomerase I Ab	< 10.0	5.9	U/mL
PR3-ANCA	< 2.0	< 1.0	U/mL
MPO-ANCA	< 3.5	< 1.0	U/mL
Anti-ARS Ab	< 25.0	< 5.0	Index
Anti-TIF1-γAb	< 32	< 5	index
Anti-Mi-2 Ab	< 53	< 5	index
Anti-MDA-5 Ab	< 32	< 4	index
AMA-M2	< 7.0	1.8	index

Ab: antibody; AMA-M2: anti-mitochondrial antibody type M2; ANCA: anti-neutrophil cytoplasmic antibody; ARS: aminoacyl-tRNA synthetase; CCP: cyclic citrullinated peptide; CH50: 50% hemolytic complement activity; ds-DNA: double-stranded DNA; Ig: immunoglobulin; MDA-5: melanoma differentiation-associated gene 5; MPO: myeloperoxidase; PR3: proteinase 3; RF: rheumatoid factor; RNP: ribonucleoprotein; Sm: Smith; SS: Sjögren’s syndrome; TIF1-γ: transcriptional intermediary factor 1-γ.

**Figure 1.**
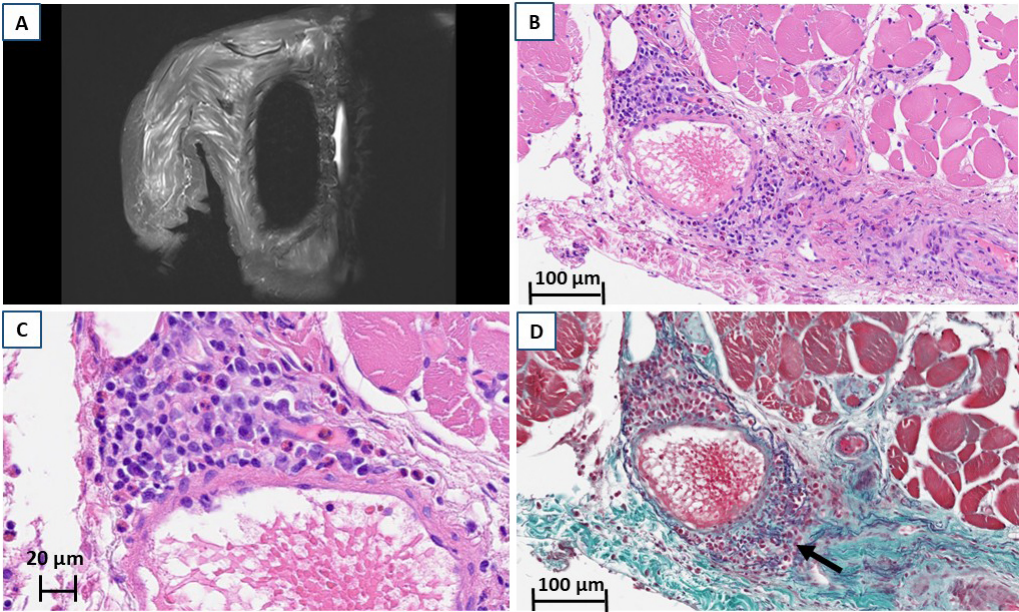
MRI and pathology. (A) MRI with short tau inversion recovery sequences of the right upper arm shows high signal intensity in the skeletal muscle. (B, C) H & E staining of the right biceps brachii reveals inflammatory cell infiltration, predominantly eosinophils and lymphocytes, within the walls of arteries. (D) Elastica Masson staining of the right biceps brachii; the arrow indicates a disruption of the external elastic lamina, with proliferation of collagen fibers surrounding the blood vessels. H & E: hematoxylin-eosin; MRI: magnetic resonance imaging.

MRI with short tau inversion recovery sequences demonstrated diffuse high-signal intensity in the muscles of both upper limbs, shoulders, and lower limbs (Figure 1A). A biopsy specimen from the right biceps brachii, identified as a high-signal area on MRI, showed inflammatory cell infiltration, predominantly eosinophils and lymphocytes, within the arterial walls (Figure 1B and 1C). Elastica Masson staining revealed disruption of the external elastic lamina and perivascular collagen fiber proliferation (Figure 1D).

She had no history of lifestyle-related disease or smoking. Based on her clinical history, fever, muscle pain, eosinophilia, MRI findings, and histology, she was diagnosed with EGPA and associated skeletal muscle involvement. To evaluate possible comorbidities, we performed a non-contrast computed tomography (CT) scan, which showed no comorbidities, including calcification in the aorta or lower-limb arteries.

Treatment with prednisolone 20 mg/day (0.5 mg/kg/day) led to rapid improvement. By day six, she was able to walk 12 m; by day 10, 30 m; and by day 12, she could ascend and descend stairs without difficulty.

## Discussion

This is the second reported case of EGPA with IC. Among the five reported cases of ANCA-associated vasculitis presenting with IC (Table 2), only one was diagnosed as EGPA ^(3)^. In the present case, histology revealed inflammatory cell infiltration in the vascular walls surrounding the skeletal muscle, suggesting ischemia due to necrotizing vasculitis.

**Table 2.** Cases of Anti-Neutrophil Cytoplasmic Antibody-Associated Vasculitis Presenting with Intermittent Claudication.

Case	Age (years)	Sex	Clinical symptoms	MPO-ANCA	PR3-ANCA	Diagnosis	Authors	Year	Publisher
Present case	80	F	Bronchial asthma, allergic rhinitis, muscle pain and weakness in the limbs, IC	Negative	Negative	EGPA	ー	ー	ー
1	69	M	Bronchial asthma, fever, IC, toe ulcers	Negative	Negative	EGPA	Inui S, Itami S, Iwai C, Yoshikawa K	2001	*J Dermatol*
2	75	M	Muscle pain in both lower legs, IC, fever, dyspnea, renal impairment	761.0 AAU	Negative	GPA	Kim MY, Bae SY, Lee M, et al	2012	*Rheumatol Int*
3	65	F	Fever, difficulty breathing, blood in the sputum, pain in both lower legs, IC	122.04 U/ml	Negative	MPA	Liao Z-H, Feng JT, Tang J-L, et al	2021	*Chin Med Sci J*
4	61	M	IC, right leg drop, muscle weakness in both legs, sensory disturbance in both legs, cerebral infarction	140 U/mL	N/A	MPA	Ando T, Watanabe H, Riku Y, et al	2023	*Eur Spine J*
5	70	M	IC, fever, purpura	32.9 U/mL	N/A	MPA	Ishizuka K, Ohira Y	2023	*BMJ Case Rep*

ANCA: anti-neutrophil cytoplasmic antibody; EGPA: eosinophilic granulomatosis with polyangiitis; F: female; GPA: granulomatosis with polyangiitis; IC: intermittent claudication; M: male; MPA: microscopic polyangiitis; MPO: myeloperoxidase; N/A: not available; PR3: proteinase 3.

IC is typically caused by peripheral arterial disease ^(4)^ or spinal canal stenosis ^(5)^ and is rarely attributed to vasculitis. In elderly patients, distinguishing EGPA-related IC from atherosclerosis is crucial. In this patient, the absence of atherosclerotic risk factors, the presence of palpable peripheral pulses, no vascular calcification on CT, and rapid and dramatic improvement with corticosteroid therapy suggested that atherosclerosis was unlikely. While non-contrast CT is not standard for evaluating peripheral vascular disease, it was used here to screen for comorbidities. We acknowledge the lack of angiography as a limitation, as small-vessel peripheral vascular disease cannot be completely ruled out. However, the rapid therapeutic response strongly supports vasculitis as the cause of IC.

Pain at rest raised the possibility of myositis; however, histopathology showed no inflammation within muscle fibers or the perimysium, ruling out true inflammatory myopathy. Muscle pain was likely due to ischemia and perivascular inflammation.

In this case, MRI played a pivotal role by identifying high-signal areas for biopsy. In a cohort study, muscle pain was reported in 40% of myeloperoxidase-ANCA-positive and 17.2% of myeloperoxidase-ANCA-negative EGPA cases ^(6)^. However, few reports have described skeletal muscle involvement in patients with elevated CK. Table 3 summarizes 11 EGPA cases with elevated CK, five of which used MRI; this is the first case in which biopsy of an MRI finding confirmed vasculitis.

**Table 3.** Cases of Skeletal Muscle Damage in EGPA with Elevated CK Levels.

Case	Age (years)	Sex	Clinical symptoms	CK (U/L)	MPO-ANCA (U/mL)	PR3-ANCA (U/mL)	MRI	Authors	Year	Publisher
Present case	80	F	Muscle pain and weakness in the limbs, intermittent claudication, BA, allergic rhinitis,	505	Negative	Negative	Diffuse high signal intensity in the muscles of both upper limbs, shoulders, and lower limbs on STIR sequences	ー	ー	ー
1	50	F	Muscle pain and weakness in both lower limbs, BA, numbness in the soles of both feet	930	N/A	N/A	N/A	Kawaguchi R, Okayama K, Handa Y, et al	1989	*Nihon Naika Gakkai Zasshi*
2	81	M	Muscle pain and weakness in both upper and lower limbs, BA, nasal polyps	538	42.1	Negative	N/A	Suresh E, Dhillon VB, Smith C, Ironside JW	2004	*J Clin Pathol*
3	57	M	Generalized muscle pain, abnormal sensation in the arm, sinusitis, chronic bronchitis	4,454	57.6	Negative	N/A	Lazzarin P, Presotto F, Polo A	2009	*Reumatismo*
4	68	F	Muscle pain and weakness in both lower limbs, fever, BA, sinusitis, hemorrhagic blisters	1,076	640	Negative	N/A	Uehara M, Hashimoto T, Sasahara E, et al	2009	*J Clin Neurosci*
5	71	M	Muscle pain and weakness in the lower limbs, edema, BA, nasal polyps	2,245	132	Negative	No abnormal findings	Tutor-Ureta P, Martín Jiménez ML, Bellas C, et al	2011	*Rev Clin Esp*
6	74	M	Generalized muscle pain, forehead claudication, fever, BA	3,708	Negative	Negative	Diffuse high signal intensity in the muscles of both lower limbs and mild muscle atrophy on STIR sequences	Parent M-E, Larue S, Ellezam B	2014	*BMC Musculoskelet Disord*
7	64	M	Muscle weakness in both lower legs, BA, sinusitis, purpura in both lower legs	2,204	168	Negative	N/A	Lim G, Lim S, Tee S-I, Ling CY	2019	*BMJ Case Rep*
8	78	M	Muscle pain and weakness in both lower limbs, fever, BA	559	Positive	N/A	Diffuse high signal intensity in the muscles of both lower limbs on T2WI	Woo K, Park YE, Jeon D, Shin JH	2020	*J Clin Neurol*
9	82	F	Muscle weakness in both upper and lower limbs, dysphagia, dyspnea	2,627	>200	N/A	Diffuse high signal intensity in the muscles of both lower limbs and mild muscle atrophy on T2WI	Koppikar S, Al-Dabie G, Jerome D, Vinik O	2020	*Rheumatol Int*
10	75	M	Muscle pain and weakness in the lower limbs, fever	632	Negative	Negative	Diffuse high signal intensity in both thigh muscles on STIR sequences	Ueta Y, Akiba Y, Yamazaki J, et al	2020	*Intern Med*
11	57	M	Muscle pain and weakness in the upper and lower limbs, sensory disturbance in both hands	1,040	> 200	Negative	N/A	Aykac S, Yuceyar AN, Akalın T, Colakoglu Z	2023	*Neurol India*

ANCA: anti-neutrophil cytoplasmic antibody; BA: bronchial asthma; CK: creatine kinase; EGPA: eosinophilic granulomatosis with polyangiitis; F: female; M: male; MPO: myeloperoxidase; MRI: magnetic resonance imaging; N/A: not available; PR3: proteinase 3; STIR: short tau inversion recovery; T2WI: T2-weighted image.

While MRI is widely used in inflammatory myopathies ^(7), (8), (9)^, its role in EGPA-associated muscle damage remains to be established.

In conclusion, EGPA should be considered in the differential diagnosis of patients presenting with IC, particularly when systemic signs such as fever, eosinophilia, and asthma are present. MRI may help detect muscle involvement, guide biopsy site selection, and facilitate timely diagnosis and treatment of EGPA-associated muscle damage.

## Article Information

### Acknowledgements

We would like to thank Editage (www.editage.jp) for English language editing.

### Authors Contributions

Conception, data acquisition, analysis, interpretation of data for the work, and drafting: Koichi Bamba. Pathology imaging, figure preparation, and interpretation of the pathology: Yuki Kamikokura. Provided substantial contribution to interpretation of data for the work and revised it critically: Daisuke Fujishiro, Tomoki Kawahata, Tsubasa Ono, Yuta Ikechi, Kitaru Tanaka, Kensaku Okamoto, Yuichi Makino, and Hiroshi Nomoto. All authors substantially contributed to the revision of the manuscript drafts. Manuscript revision and final approval: All authors.

### Conflicts of Interest

None

### IRB Approval Code and Name of the Institution

According to the guidelines of the Institutional Review Board of Asahikawa Medical University, formal ethical approval was not required for a single case report.

### Informed Consent

Written informed consent for publication was obtained from the patient.
